# 2-Eth­oxy­ethyl (*Z*)-2-cyano-3-[(*N*-phenyl­carbamo­yl)amino]­prop-2-enoate

**DOI:** 10.1107/S1600536811055000

**Published:** 2012-01-07

**Authors:** Shihua Zhong, Dongmei Wei, Jianbing Liu, Bingyu Liu

**Affiliations:** a College of Chemistry and Chemical Engineering, Hunan Normal University, Changsha 410081, Hunan, People’s Republic of China

## Abstract

The crystal structure of the title compound, C_15_H_17_N_3_O_4_, is stabilized by inter­molecular N—H⋯N hydrogen bonds. An intra­molecular N—H⋯O hydrogen bond also occurs.

## Related literature

The title compound was synthesized as a possible novel herbicide. For details of the synthesis, see: Wang *et al.* (2004 ▶); Senda *et al.* (1972 ▶). For reviews of cyano­acrylate derivatives as bioactive agents, see: Zhang *et al.* (2008 ▶); Liu *et al.* (1998 ▶).
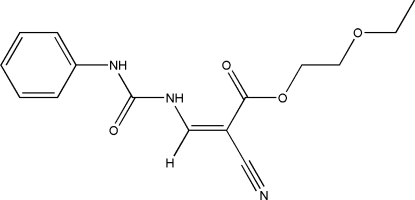


## Experimental

### 

#### Crystal data

C_15_H_17_N_3_O_4_
*M* *_r_* = 303.32
Monoclinic, 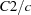

*a* = 25.102 (7) Å
*b* = 12.013 (3) Å
*c* = 10.436 (3) Å
β = 96.248 (4)°
*V* = 3128.4 (16) Å^3^
*Z* = 8
Mo *K*α radiation
μ = 0.10 mm^−1^
*T* = 153 K
0.48 × 0.44 × 0.09 mm

#### Data collection

Rigaku AFC10/Saturn724+ diffractometer
Absorption correction: multi-scan (*ABSCOR*; Higashi, 1995 ▶) *T*_min_ = 0.956, *T*_max_ = 0.992
16240 measured reflections
4149 independent reflections
2994 reflections with *I* > 2σ(*I*)
*R*_int_ = 0.038

#### Refinement

*R*[*F* ^2^ > 2σ(*F* ^2^)] = 0.043
*wR*(*F* ^2^) = 0.116
*S* = 1.00
4149 reflections
208 parameters
H atoms treated by a mixture of independent and constrained refinement
Δρ_max_ = 0.28 e Å^−3^
Δρ_min_ = −0.18 e Å^−3^

### 

Data collection: *CrystalClear* (Rigaku, 2008 ▶); cell refinement: *CrystalClear*; data reduction: *CrystalClear*; program(s) used to solve structure: *SHELXS97* (Sheldrick, 2008 ▶); program(s) used to refine structure: *SHELXL97* (Sheldrick, 2008 ▶); molecular graphics: *SHELXTL* (Sheldrick, 2008 ▶); software used to prepare material for publication: *SHELXL97*.

## Supplementary Material

Crystal structure: contains datablock(s) I, global. DOI: 10.1107/S1600536811055000/hg5148sup1.cif

Structure factors: contains datablock(s) I. DOI: 10.1107/S1600536811055000/hg5148Isup2.hkl

Supplementary material file. DOI: 10.1107/S1600536811055000/hg5148Isup3.cml

Additional supplementary materials:  crystallographic information; 3D view; checkCIF report

## Figures and Tables

**Table 1:** Hydrogen-bond geometry (Å, °)

*D*—H⋯*A*	*D*—H	H⋯*A*	*D*⋯*A*	*D*—H⋯*A*
N2—H2*N*⋯O3	0.910 (15)	2.068 (15)	2.7543 (14)	131.2 (12)
N1—H1*N*⋯N3^i^	0.867 (15)	2.050 (15)	2.9120 (15)	172.6 (14)

Symmetry code: (i) 
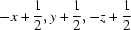
.

**Fig. 1.**
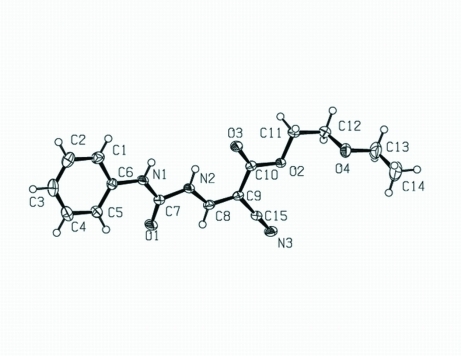
The molecular structure of compound shows displacement ellipsoids drawn at the 50% probability level, all H atoms have been omitted for clarity.

**Fig. 2.**
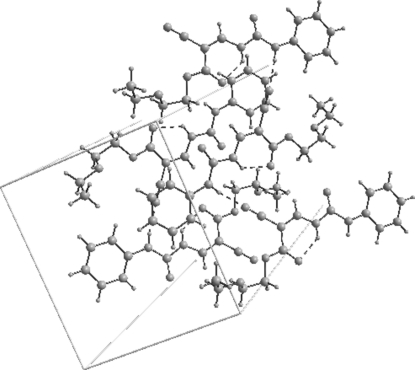
The crystal packing of the title compound with hydrogen bonds drawn as dashed lines.

## supplementary crystallographic information

### Comment

Previous studies have shown that cyanoacrylate derivatives are an important class of compounds with high bioactivities and can be applied as herbicide (Zhang *et al.*, 2008), urea derivatives also exhibit good herbicidal activities (Liu *et al.*, 1998), both kinds of compounds are inhibitors of photosystem II electron transport and inhibit the growth of weeds by disrupting photosynthetic electron transport. A novel cyanoacrylate compound (C_15_H_17_N_3_O_4_) which bears a phenyl urea unit was synthesized and investigated for its ability to inhibit PSII electron transport, its crystal structure is reported here.

The crystal structure of title compound is stabilized by inter-molecular N—H···N hydrogen bonds, the orientation of phenylurea and ester carbonyl is *cis* and an intramolecular N—H···O hydrogen bond was generated to stabilize the conformation.

### Experimental

The title compound was prepared according to the reported method (Wang *et al.*, 2004; Senda *et al.*, 1972). A mixture of 2-ethoxyethyl cyanoacetate (0.55 g, 3.5 mmol), Phenylurea (0.39 g 2.9 mmol) and triethyl orthoformate (0.59 ml, 3.5 mmol) was heated at 378 K for 2 hr, cooled to room temperature, the precipitation was filtered off, washed with hexane and recrystallized from ethanol to give white solid (yield 37%), mp: 458 K. Crystals of (I) suitable for XRD were obtained by slow evaporation of a mixture solution of ethanol and acetone in a ratio of 1:2 at 293 K.

### Refinement

Positional parameters of carbon H atoms were calculated geometically and were allowed to ride on the C atoms to which they are bonded, with C—H = 0.95 to 0.99 Å, *U*_iso_(H) = 1.2 or 1.5 *U*_eq_(C). The H atoms of the N atoms were located in difference Fourier maps and refined freely.

### Crystal data

**Table:**

C_15_H_17_N_3_O_4_	*F*(000) = 1280
*M**_r_* = 303.32	*D*_x_ = 1.288 Mg m^−^^3^
Monoclinic, *C*2/*c*	Mo *K*α radiation, λ = 0.71073 Å
Hall symbol: -C 2yc	Cell parameters from 4353 reflections
*a* = 25.102 (7) Å	θ = 2.7–29.1°
*b* = 12.013 (3) Å	µ = 0.10 mm^−^^1^
*c* = 10.436 (3) Å	*T* = 153 K
β = 96.248 (4)°	Platelet, colorless
*V* = 3128.4 (16) Å^3^	0.48 × 0.44 × 0.09 mm
*Z* = 8	

### Data collection

**Table:**

Rigaku AFC10/Saturn724+ diffractometer	4149 independent reflections
Radiation source: Rotating Anode	2994 reflections with *I* > 2σ(*I*)
graphite	*R*_int_ = 0.038
Detector resolution: 28.5714 pixels mm^-1^	θ_max_ = 29.1°, θ_min_ = 2.7°
phi and ω scans	*h* = −34→32
Absorption correction: multi-scan (*ABSCOR*; Higashi, 1995)	*k* = −16→16
*T*_min_ = 0.956, *T*_max_ = 0.992	*l* = −13→14
16240 measured reflections	

### Refinement

**Table:**

Refinement on *F*^2^	Primary atom site location: structure-invariant direct methods
Least-squares matrix: full	Secondary atom site location: difference Fourier map
*R*[*F*^2^ > 2σ(*F*^2^)] = 0.043	Hydrogen site location: inferred from neighbouring sites
*wR*(*F*^2^) = 0.116	H atoms treated by a mixture of independent and constrained refinement
*S* = 1.00	*w* = 1/[σ^2^(*F*_o_^2^) + (0.0616*P*)^2^ + 0.136*P*] where *P* = (*F*_o_^2^ + 2*F*_c_^2^)/3
4149 reflections	(Δ/σ)_max_ < 0.001
208 parameters	Δρ_max_ = 0.28 e Å^−^^3^
0 restraints	Δρ_min_ = −0.17 e Å^−^^3^

### Special details

**Table:**

Geometry. All e.s.d.'s (except the e.s.d. in the dihedral angle between two l.s. planes) are estimated using the full covariance matrix. The cell e.s.d.'s are taken into account individually in the estimation of e.s.d.'s in distances, angles and torsion angles; correlations between e.s.d.'s in cell parameters are only used when they are defined by crystal symmetry. An approximate (isotropic) treatment of cell e.s.d.'s is used for estimating e.s.d.'s involving l.s. planes.
Refinement. Refinement of *F*^2^ against ALL reflections. The weighted *R*-factor *wR* and goodness of fit *S* are based on *F*^2^, conventional *R*-factors *R* are based on *F*, with *F* set to zero for negative *F*^2^. The threshold expression of *F*^2^ > σ(*F*^2^) is used only for calculating *R*-factors(gt) *etc*. and is not relevant to the choice of reflections for refinement. *R*-factors based on *F*^2^ are statistically about twice as large as those based on *F*, and *R*- factors based on ALL data will be even larger.

### Fractional atomic coordinates and isotropic or equivalent isotropic displacement parameters (Å^2^)

**Table:**

	*x*	*y*	*z*	*U*_iso_*/*U*_eq_	
O1	0.15659 (4)	0.22114 (7)	0.47309 (9)	0.0364 (2)	
O2	0.32432 (3)	0.36646 (7)	0.09862 (8)	0.0255 (2)	
O3	0.27763 (4)	0.46231 (7)	0.23556 (8)	0.0306 (2)	
O4	0.42663 (4)	0.39232 (8)	−0.00208 (9)	0.0364 (2)	
N1	0.16467 (4)	0.40528 (8)	0.53128 (10)	0.0250 (2)	
N2	0.21226 (4)	0.33631 (8)	0.37397 (9)	0.0240 (2)	
N3	0.28465 (4)	0.09868 (8)	0.07213 (10)	0.0289 (3)	
C1	0.11615 (5)	0.52205 (11)	0.66349 (12)	0.0307 (3)	
H1	0.1298	0.5844	0.6216	0.037*	
C2	0.08347 (6)	0.53787 (13)	0.76109 (13)	0.0375 (3)	
H2	0.0747	0.6112	0.7858	0.045*	
C3	0.06367 (6)	0.44733 (14)	0.82240 (13)	0.0396 (3)	
H3	0.0416	0.4583	0.8896	0.047*	
C4	0.07616 (5)	0.34066 (13)	0.78556 (13)	0.0362 (3)	
H4	0.0625	0.2785	0.8278	0.043*	
C5	0.10845 (5)	0.32346 (11)	0.68761 (12)	0.0282 (3)	
H5	0.1165	0.2500	0.6622	0.034*	
C6	0.12894 (5)	0.41423 (10)	0.62707 (11)	0.0232 (3)	
C7	0.17519 (5)	0.31405 (9)	0.46344 (12)	0.0242 (3)	
C8	0.22843 (5)	0.25503 (9)	0.29863 (11)	0.0227 (3)	
H8	0.2142	0.1827	0.3086	0.027*	
C9	0.26364 (5)	0.26801 (9)	0.20886 (11)	0.0221 (2)	
C10	0.28862 (5)	0.37541 (10)	0.18432 (11)	0.0229 (3)	
C11	0.34591 (5)	0.47118 (10)	0.05780 (13)	0.0291 (3)	
H11A	0.3684	0.5062	0.1306	0.035*	
H11B	0.3164	0.5229	0.0284	0.035*	
C12	0.37881 (5)	0.44736 (11)	−0.05006 (12)	0.0306 (3)	
H12A	0.3581	0.4000	−0.1153	0.037*	
H12B	0.3876	0.5179	−0.0921	0.037*	
C13	0.45942 (7)	0.36769 (16)	−0.10075 (16)	0.0567 (5)	
H13A	0.4701	0.4375	−0.1414	0.068*	
H13B	0.4393	0.3214	−0.1680	0.068*	
C14	0.50822 (8)	0.30642 (17)	−0.0432 (2)	0.0745 (6)	
H14A	0.5255	0.3486	0.0303	0.112*	
H14B	0.5333	0.2978	−0.1082	0.112*	
H14C	0.4978	0.2328	−0.0140	0.112*	
C15	0.27583 (5)	0.17378 (9)	0.13389 (11)	0.0225 (2)	
H2N	0.2248 (6)	0.4059 (13)	0.3597 (14)	0.038 (4)*	
H1N	0.1787 (6)	0.4663 (13)	0.5061 (13)	0.035 (4)*	

### Atomic displacement parameters (Å^2^)

**Table:**

	*U*^11^	*U*^22^	*U*^33^	*U*^12^	*U*^13^	*U*^23^
O1	0.0471 (6)	0.0169 (4)	0.0493 (6)	−0.0065 (4)	0.0241 (5)	−0.0041 (4)
O2	0.0295 (5)	0.0150 (4)	0.0342 (5)	0.0004 (3)	0.0134 (4)	0.0003 (3)
O3	0.0400 (5)	0.0168 (4)	0.0375 (5)	−0.0024 (4)	0.0153 (4)	−0.0060 (4)
O4	0.0314 (5)	0.0425 (6)	0.0375 (5)	0.0078 (4)	0.0141 (4)	0.0064 (4)
N1	0.0334 (6)	0.0155 (5)	0.0278 (5)	−0.0017 (4)	0.0116 (5)	0.0003 (4)
N2	0.0296 (6)	0.0155 (5)	0.0285 (5)	−0.0011 (4)	0.0100 (4)	−0.0003 (4)
N3	0.0372 (6)	0.0178 (5)	0.0324 (6)	0.0009 (4)	0.0075 (5)	−0.0013 (4)
C1	0.0371 (7)	0.0251 (6)	0.0304 (7)	0.0022 (5)	0.0059 (6)	−0.0027 (5)
C2	0.0379 (8)	0.0414 (8)	0.0337 (7)	0.0084 (6)	0.0059 (6)	−0.0101 (6)
C3	0.0309 (7)	0.0592 (10)	0.0302 (7)	0.0019 (7)	0.0106 (6)	−0.0055 (7)
C4	0.0303 (7)	0.0464 (9)	0.0330 (7)	−0.0043 (6)	0.0084 (6)	0.0058 (6)
C5	0.0283 (7)	0.0285 (6)	0.0283 (6)	−0.0019 (5)	0.0057 (5)	0.0028 (5)
C6	0.0235 (6)	0.0252 (6)	0.0212 (6)	0.0009 (4)	0.0031 (5)	−0.0007 (5)
C7	0.0271 (6)	0.0182 (6)	0.0282 (6)	0.0003 (4)	0.0070 (5)	0.0011 (5)
C8	0.0265 (6)	0.0161 (5)	0.0256 (6)	0.0003 (4)	0.0030 (5)	−0.0002 (4)
C9	0.0259 (6)	0.0146 (5)	0.0261 (6)	0.0014 (4)	0.0045 (5)	−0.0008 (4)
C10	0.0254 (6)	0.0181 (6)	0.0258 (6)	0.0011 (4)	0.0049 (5)	−0.0009 (4)
C11	0.0336 (7)	0.0165 (6)	0.0391 (7)	−0.0024 (5)	0.0123 (6)	0.0018 (5)
C12	0.0333 (7)	0.0263 (6)	0.0337 (7)	0.0011 (5)	0.0101 (6)	0.0050 (5)
C13	0.0563 (10)	0.0660 (12)	0.0536 (10)	0.0232 (9)	0.0333 (8)	0.0157 (8)
C14	0.0570 (12)	0.0897 (16)	0.0838 (14)	0.0342 (11)	0.0399 (11)	0.0275 (12)
C15	0.0255 (6)	0.0172 (5)	0.0251 (6)	0.0001 (4)	0.0046 (5)	0.0030 (4)

### Geometric parameters (Å, °)

**Table:**

O1—C7	1.2183 (14)	C4—C5	1.3875 (18)
O2—C10	1.3376 (14)	C4—H4	0.9500
O2—C11	1.4520 (14)	C5—C6	1.3869 (17)
O3—C10	1.2181 (14)	C5—H5	0.9500
O4—C12	1.4135 (15)	C8—C9	1.3650 (16)
O4—C13	1.4177 (17)	C8—H8	0.9500
N1—C7	1.3465 (15)	C9—C15	1.4281 (16)
N1—C6	1.4179 (15)	C9—C10	1.4691 (16)
N1—H1N	0.867 (15)	C11—C12	1.4943 (18)
N2—C8	1.3439 (15)	C11—H11A	0.9900
N2—C7	1.4136 (15)	C11—H11B	0.9900
N2—H2N	0.910 (15)	C12—H12A	0.9900
N3—C15	1.1443 (15)	C12—H12B	0.9900
C1—C2	1.3887 (18)	C13—C14	1.498 (2)
C1—C6	1.3970 (17)	C13—H13A	0.9900
C1—H1	0.9500	C13—H13B	0.9900
C2—C3	1.382 (2)	C14—H14A	0.9800
C2—H2	0.9500	C14—H14B	0.9800
C3—C4	1.384 (2)	C14—H14C	0.9800
C3—H3	0.9500		
			
C10—O2—C11	115.17 (9)	C8—C9—C15	118.61 (11)
C12—O4—C13	112.22 (11)	C8—C9—C10	122.71 (10)
C7—N1—C6	127.43 (10)	C15—C9—C10	118.66 (10)
C7—N1—H1N	114.8 (10)	O3—C10—O2	124.24 (11)
C6—N1—H1N	117.3 (10)	O3—C10—C9	123.70 (11)
C8—N2—C7	120.76 (10)	O2—C10—C9	112.06 (10)
C8—N2—H2N	116.1 (9)	O2—C11—C12	108.04 (10)
C7—N2—H2N	123.0 (9)	O2—C11—H11A	110.1
C2—C1—C6	119.87 (13)	C12—C11—H11A	110.1
C2—C1—H1	120.1	O2—C11—H11B	110.1
C6—C1—H1	120.1	C12—C11—H11B	110.1
C3—C2—C1	120.20 (13)	H11A—C11—H11B	108.4
C3—C2—H2	119.9	O4—C12—C11	109.89 (10)
C1—C2—H2	119.9	O4—C12—H12A	109.7
C2—C3—C4	119.78 (13)	C11—C12—H12A	109.7
C2—C3—H3	120.1	O4—C12—H12B	109.7
C4—C3—H3	120.1	C11—C12—H12B	109.7
C3—C4—C5	120.72 (13)	H12A—C12—H12B	108.2
C3—C4—H4	119.6	O4—C13—C14	108.98 (14)
C5—C4—H4	119.6	O4—C13—H13A	109.9
C6—C5—C4	119.60 (13)	C14—C13—H13A	109.9
C6—C5—H5	120.2	O4—C13—H13B	109.9
C4—C5—H5	120.2	C14—C13—H13B	109.9
C5—C6—C1	119.82 (12)	H13A—C13—H13B	108.3
C5—C6—N1	123.78 (11)	C13—C14—H14A	109.5
C1—C6—N1	116.35 (11)	C13—C14—H14B	109.5
O1—C7—N1	127.11 (12)	H14A—C14—H14B	109.5
O1—C7—N2	120.92 (11)	C13—C14—H14C	109.5
N1—C7—N2	111.98 (10)	H14A—C14—H14C	109.5
N2—C8—C9	125.30 (11)	H14B—C14—H14C	109.5
N2—C8—H8	117.3	N3—C15—C9	178.57 (13)
C9—C8—H8	117.3		
			
C6—C1—C2—C3	0.1 (2)	C7—N2—C8—C9	179.49 (11)
C1—C2—C3—C4	−0.5 (2)	N2—C8—C9—C15	−178.46 (11)
C2—C3—C4—C5	0.1 (2)	N2—C8—C9—C10	0.22 (19)
C3—C4—C5—C6	0.8 (2)	C11—O2—C10—O3	−6.56 (17)
C4—C5—C6—C1	−1.16 (18)	C11—O2—C10—C9	172.90 (10)
C4—C5—C6—N1	176.29 (11)	C8—C9—C10—O3	−4.72 (19)
C2—C1—C6—C5	0.71 (19)	C15—C9—C10—O3	173.95 (11)
C2—C1—C6—N1	−176.93 (11)	C8—C9—C10—O2	175.81 (10)
C7—N1—C6—C5	15.9 (2)	C15—C9—C10—O2	−5.52 (16)
C7—N1—C6—C1	−166.53 (12)	C10—O2—C11—C12	−172.26 (10)
C6—N1—C7—O1	−1.3 (2)	C13—O4—C12—C11	180.00 (13)
C6—N1—C7—N2	178.81 (11)	O2—C11—C12—O4	−71.42 (13)
C8—N2—C7—O1	−0.93 (18)	C12—O4—C13—C14	−178.18 (14)
C8—N2—C7—N1	178.97 (10)		

### Hydrogen-bond geometry (Å, °)

**Table:**

*D*—H···*A*	*D*—H	H···*A*	*D*···*A*	*D*—H···*A*
N2—H2N···O3	0.910 (15)	2.068 (15)	2.7543 (14)	131.2 (12)
N1—H1N···N3^i^	0.867 (15)	2.050 (15)	2.9120 (15)	172.6 (14)

Symmetry codes: (i) −*x*+1/2, *y*+1/2, −*z*+1/2.
